# Patient perspectives on chemotherapy de‐escalation in breast cancer

**DOI:** 10.1002/cam4.3891

**Published:** 2021-05-01

**Authors:** Gabrielle B. Rocque, Courtney P. Williams, Courtney Andrews, Timothy C. Childers, Kimberly D. Wiseman, Kathleen Gallagher, Nadine Tung, Alan Balch, Valerie M Lawhon, Stacey A Ingram, Thelma Brown, Tara Kaufmann, Mary L. Smith, Angela DeMichele, Antonio C. Wolff, Lynne Wagner

**Affiliations:** ^1^ O’Neal Comprehensive Cancer Center University of Alabama at Birmingham Birmingham AL USA; ^2^ Division of Hematology and Oncology Department of Medicine University of Alabama at Birmingham Birmingham AL USA; ^3^ Department of Medicine Division of Geriatrics, Gerontology, and Palliative Care University of Alabama at Birmingham Birmingham AL USA; ^4^ UAB College of Arts and Sciences School of Anthropology University of Alabama at Birmingham Birmingham AL USA; ^5^ Wake Forest School of Medicine Winston‐Salem NC USA; ^6^ Patient Advocate Foundation Hampton VA USA; ^7^ Beth Israel Deaconess Medical Center Boston MA USA; ^8^ Dell Medical School University of Texas at Austin LiveSTRONG Cancer Institute Austin TX USA; ^9^ ECOG‐ACRIN Cancer Research Advocates Committee Philadelphia PA USA; ^10^ Abramson Cancer Center University of Pennsylvania Philadelphia PA USA; ^11^ John Hopkins University Baltimore MD USA

**Keywords:** de‐escalation, deimplementation, patient perspectives, recurrence distress

## Abstract

**Background:**

Given excellent survival outcomes in breast cancer, there is interest in de‐escalating the amount of chemotherapy delivered to patients. This approach may be of even greater importance in the setting of the COVID‐19 pandemic.

**Methods:**

This concurrent mixed methods study included (1) interviews with patients and patient advocates and (2) a cross‐sectional survey of women with breast cancer served by a charitable nonprofit organization. Questions evaluated interest in de‐escalation trial participation, perceived barriers/facilitators to participation, and language describing de‐escalation.

**Results:**

Sixteen patient advocates and 24 patients were interviewed. Key barriers to de‐escalation included fear of recurrence, worry about decision regret, lack of clinical trial interest, and dislike for focus on less treatment. Facilitators included trust in physician recommendation, toxicity avoidance, monitoring for progression, perception of good prognosis, and impact on daily life. Participants reported that the COVID‐19 pandemic made them more likely to avoid chemotherapy if possible. Of 91 survey respondents, many (43%) patients would have been unwilling to participation in a de‐escalation clinical trial. The most commonly reported barrier to participation was fear of recurrence (85%). Few patients (19%) considered clinical trials themselves as a barrier to de‐escalation trial participation. The most popular terminology describing chemotherapy de‐escalation was “lowest effective chemotherapy dose” (53%); no patients preferred the term “de‐escalation.”

**Conclusions:**

Fear of recurrence is a common concern among breast cancer survivors and patient advocates, contributing to resistance to de‐escalation clinical trial participation. Additional research is needed to understand how to engage patients in de‐escalation trials.

## Graphical abstract

Over 40% of patients would not be interested in participating in clinical trials testing de‐escalation, with the most common barriers being fear of recurrence. Common facilitators included toxicity avoidance, trust in the physician, and use of patient‐centric language to describe the trial.

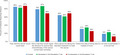

## 1 INTRODUCTION

Clinical trials in breast cancer have historically focused on improving survival by the *addition* of chemotherapeutic agents to existing treatment regimens. However, new availability of effective targeted therapies and improved prognostic markers have created an opportunity to shift this paradigm to avoid overtreatment by *reducing*, or de‐escalating, traditional chemotherapy where limited benefit exists.^1^ For example, in human epidermal growth factor receptor 2 (HER2)‐positive breast cancer, the APHINITY trial demonstrated that adding pertuzumab to standard adjuvant, combination chemotherapy improved 3‐year invasive disease‐free survival from 93% to 94%. However, this improvement had marginal clinical relevance and was associated with substantial toxicities and cost, prompting a study (COMPASSHER2) of single‐agent chemotherapy with these targeted agents.^2^ This study will complement previous trials in breast cancer testing less treatment, such as the Cancer and Leukemia Group B (CALGB) 9434 trial omitting radiation following lumpectomy in older adults with breast cancer^3^ and use of single‐agent paclitaxel with trastuzumab (TH) instead of combination chemotherapy for patients with small, node‐negative breast cancer.^4^ Furthermore, not all de‐escalation strategies will result in outcomes equivalent to standard of care. In recent Phase III trials treating oropharyngeal cancer, investigators withheld radiation to spare toxicity but noted inferior survival for the de‐escalation arms.^5^, ^6^ Therefore, in order to identify the option which both minimizes toxicity and maintains survival, rigorous testing of de‐escalation strategies is needed.

Experience with de‐escalation trials in other tumor types has shown that patient enrollment is a particular challenge. PIVOT randomized patients with prostate cancer to radical prostatectomy versus observation. It required 8 years (1994–2004) and 52 sites for enrollment since only 15% (731/5023) of eligible patients agreed to participate.^7^ Limited data exist on reasons for patient reluctance to enroll in or complete de‐escalation trials. Furthermore, perspectives on chemotherapy de‐escalation may be actively changing in the setting of the ongoing COVID‐19 pandemic. Early case fatality rates suggested increased risk of death or serious events from COVID‐19 for patients with active cancer compared to patients without cancer.^8^, ^9^, ^10^ This is supported by further analysis by Wang and colleagues, in which patients with a recent cancer had higher death rates than the general adult population (15% vs. 5.6%).^11^ We sought to understand patient perspectives regarding breast cancer de‐escalation trials, identify perceived barriers and facilitators to participation, and understand how the COVID‐19 pandemic influences these views through interviews conducted both before and during the pandemic.

## 2 METHODS

### 2.1 Study design and sample

This convergent, synergistic mixed methods study evaluated patient perspectives surrounding chemotherapy de‐escalation. This design uses qualitative and quantitative components conducted and analyzed simultaneously to provide complimentary information on a topic for deeper understanding. Qualitative interview data were collected from patients with breast cancer and patient advocates. Quantitative survey data were collected from a nationwide sample of women with breast cancer.

### 2.2 Qualitative component

#### 2.2.1 Participant sample

Medical oncology clinic lists were reviewed from October 2019 to May 2020 to identify eligible women with stage II–III breast cancer to target patients who would be appropriate for chemotherapy. The sample was enriched for patients with HER2+ early stage breast cancer (EBC) to inform planning for a future HER2+ trial. Exclusion criteria included: age <18 years old; inability to read or speak English; inability to provide informed consent; or deemed inappropriate for interview by their medical oncologist. Eligible patients were approached about interview participation by a member of the study team with permission from the treating oncologist. A convenience sample of patient advocates was identified through advocacy organizations (ECOG‐ACRIN Cancer Research Advocates Committee, Translational Breast Cancer Research Consortium, Patient Advocate Foundation [PAF], CancerCare, Forge) and invited by e‐mail to participate. Some, but not all, patient advocates were breast cancer survivors. Written and electronically signed informed consent was obtained for all participating patients and advocates. Patient participants were incentivized with a $50 gift card at interview completion.

#### 2.2.2 Data collection

Participants completed a demographic questionnaire prior to the interview which collected age, race, education, marital status, geographic residence, and decision‐making preference (Control Preferences Scale^8^). For patients, details of breast cancer diagnosis and treatment were abstracted from the medical record. Semi‐structured interviews were conducted by a medical oncologist with training in qualitative research (GR) in person or by phone. Of note, no patient had their treatment decision made by the interviewing oncologist. Following an explanation of the de‐escalation concept and rationale (Supporting Information, Document 1), interview questions explored patient interest in de‐escalation study participation, barriers and facilitators to participation, preferred verbiage to describe the concept of de‐escalation, and recommendations for future patient engagement. Questions eliciting participant perspectives surrounding the potential impact of COVID‐19 on de‐escalation were added for interviews conducted after March 2020. These questions were asked at the end of the interview to evaluate for changes in perspective on de‐escalation related to the pandemic.

#### 2.2.3 Analysis

Using qualitative content analysis,^12^ three independent coders with medical anthropology, public health, and medical expertise (CA, KW, and CC) developed an open coding scheme.^13^ The final coding schema was reviewed and finalized by the multidisciplinary team, which included three primary coders (CA, KW, and CC), an oncologist (GR), and a psychologist (LW). Two primary coders with medical anthropology and psychology expertise (CA and KW) subsequently used NVivo software (QRS International) and Atlas.ti (Version 8) to conduct “focused coding,” which included a detailed analysis of themes identified during open coding. Discrepancies were resolved by a third coder (CC). The process was repeated until thematic saturation was reached.^14^ Overarching themes pertaining to treatment de‐escalation were identified, exemplary quotes characterizing themes were highlighted, and investigator insights relevant to the decision‐making process were noted in memo format. Descriptive statistics were calculated for patient demographic data and the frequency of specific themes.

### 2.3 Quantitative component

#### 2.3.1 Patient sample

Cross‐sectional survey data on patients with early stage (I–III) or metastatic (stage IV) breast cancer from November to December 2019 were collected by PAF charitable nonprofit organization that provides financial support to patients. Exclusion criteria included women <18 years old, unable to read English, without a valid e‐mail address, and those not wishing to be contacted by PAF. Women were sent an e‐mail with a customized link inviting them to complete the confidential survey (Supporting Information, Document 2). Survey questions were created de novo based on early qualitative findings. Questions pertaining to preferred language included verbiage from qualitative respondents. Surveys were pretested with patient advocates, physicians, and experts in patient‐reported outcome measures to assess face validity. Informed consent was obtained electronically before survey initiation. Respondents who initiated the survey were incentivized with a $10 digital gift card. Respondents answered five questions regarding de‐escalation as part of a 41‐question survey on decision‐making.

#### 2.3.2 Analysis

Mean differences, or effect sizes, were calculated using Cohen's *d* (continuous variables) or Cramer's *V* (categorical variables) to determine the magnitude of relationships in bivariate associations. For Cohen's *d*, an effect size of 0.2 is considered a small effect, 0.5 a medium effect, and 0.8 a large effect, while for Cramer's *V*, an effect size of 0.1 is considered a small effect, 0.3 a medium effect, and 0.5 a large effect when comparing across two categories.^15^ Risk of de‐escalation trial disinterest was estimated using risk ratios (RR) and corresponding 95% confidence intervals (CIs) from a generalized linear model with a log link and Poisson distribution with robust variance estimates. This exploratory model included age, race, marital status, employment status, income, cancer diagnosis stage, recurrence fear, and decision regret as covariables. A sensitivity analysis assessing only patients with EBC without a recurrence was conducted. Data collection was conducted using Sawtooth Software Version 9.6.1, and data analysis was completed using SAS^©^ software, version 9.4 (SAS Institute).

### 2.4 Mixed methods

Data from qualitative and quantitative components were compared side‐by‐side to triangulate results and provide an analysis of similar and divergent patient and advocate perspectives.

## 3 RESULTS

### 3.1 Qualitative results

#### 3.1.1 Participants

Forty women participated in qualitative interviews (24 patients, 16 patient advocates; Table 1). The median age of patients was 57 years (range 33–79); 13% of patient advocates and 42% of patients were Black or African American. Most participants preferred a shared decision‐making approach to treatment decisions.

**TABLE 1:** Qualitative interview participant demographics and clinical characteristics (*N* = 40)

	Patients	Advocates
	*n* = 24	*n* = 16
	*n* (%)	*n* (%)
Age (median, IQR)	57	61
Median (range)	33–79	36–75
Race		
Black or African American	10 (41.7)	2 (12.5)
White	14 (58.3)	14 (87.5)
Highest level of education		
High school	5 (20.8)	0 (0.0)
Some college/vocational or technical school	9 (37.5)	2 (12.6)
College graduate (4 year)	7 (29.2)	5 (31.3)
Master's degree or professional degree	2 (8.3)	8 (50.0)
Other	1 (4.2)	1 (6.3)
Marital status		
Single, unmarried, living with significant other	5 (20.8)	2 (12.5)
Married	14 (58.3)	13 (81.3)
Divorced/separated/widowed	5 (20.8)	1 (6.3)
Preference for treatment decision‐making style (Control Preferences Scale)		
Patient‐driven decision‐making	1 (4.2)	0 (0.0)
Patient‐driven decision‐making with provider input	5 (20.8)	9 (56.3)
Shared decision‐making	14 (58.3)	6 (37.5)
Physician‐driven decision‐making with patient input	2 (8.3)	1 (6.3)
Physician‐driven decision‐making	1 (4.2)	0 (0.0)
Missing	1 (4.2)	0 (0.0)
Breast cancer diagnosis stage		
II	16 (66.7)	—
III	8 (33.3)	—
Breast cancer type		
ER/PR+HER2−	8 (33.3)	—
ER/PR−HER2+	11 (45.8)	—
ER/PR−HER2−	5 (20.8)	—

Abbreviations: ER, estrogen receptor; HER2, human epidermal growth factor receptor 2; IQR, interquartile range; PR, progesterone receptor.

#### 3.1.2 Barriers

Participants identified several barriers to participation in clinical trials of reduced chemotherapy (Table 2). The primary barrier was fear that “*it wouldn't* *get it all*.” Fourteen patients (58%) and 11 advocates (69%) mentioned the fear of recurrence and inefficacy as a major hurdle in patients’ willingness to pursue less aggressive chemotherapy regimens. One advocate reiterated the compulsion to do “*whatever it takes at that time to make sure that you've covered all your bases and you've got the right treatment*.” Participants recognized that recurrence would impact survival, saying “*we need to do everything we can so it doesn't come back because we know if it comes back then it's treatable but not curable*. *This is the time of cure*.” Another source of reluctance was lack of study data on efficacy, saying “*Ooh, ‘study,’ that means they don't know the answer,* *yet*.” This barrier may be particularly problematic for Black women, with one participant discussing the lingering negative impact of the U.S. Public Health Service Syphilis Study at Tuskegee, a study that withheld syphilis treatment from research participants in order to study progression of disease.^16^

**TABLE 2:** Barriers to de‐escalation trial participation

Barrier	Exemplar quotes
Fear of recurrence/inefficacy	“The main thing is you want to be sure that it's gone and that it doesn't come back.” (Patient) “We need to do everything we can so it doesn't come back, because we know if it comes back then it's treatable but not curable.” (Advocate)
Dislike for focus on less treatment	“I guess it's better safe than sorry.” (Patient) “Well, why wouldn't you do every single thing you could possibly do that was even remotely in your control?” (Advocate)
Reluctance to participate in clinical trial	“I want to know I’m not being played with, and I’m not being just kind of a guinea pig.” (Patient) “[Patients] don't like new, they want the old trusty, what works good and that kind of thing.” (Advocate)
Lack of data/fear of unknown	“Ooh, ‘study,’ that means they don't know the answers yet.” (Patient) “Numbers have great power.” (Advocate)
Age/family situation	“In my mind, I had to be able to look my kids in the eyes and know I did everything in my power. So if there wasn't research to support the lower dose at that age, I wouldn't have done it.” (Patient) “They (patients with young children) can feel like they did everything that they possibly could have. They left no stone unturned.” (Advocate)
Fear of regret	“I know that's a fine line, because chemotherapy has its own issues and stuff, but I'm more of a person where I would just want to go ahead and get as much treatment as I possibly can that is recommended, because I don't want to look back and say I should have had more.” (Patient) “I think people are afraid that they will regret their decision, right? You don't really know if it's right or wrong.” (Advocate)
Perception of lower risk	“What drove that decision for me? I had a really, I had a really good prognosis, and I had the experts in the field telling me, ‘It's fine. You don't need to do anything more.’” (Patient) “So I believe that sort of teaching people about risk and helping them to understand how to balance risk is an extremely, extremely important thing to be able to do…But you really have to spend time talking to people about, ‘We put you in a low risk group because we understand that for patients who have no evidence of disease at this point, their risk of recurrence is only Y.’" (Advocate)

#### 3.1.3 Facilitators

Several patients (58%) would have been interested in a less aggressive approach when initially making decisions about cancer treatment. Avoiding physical toxicities was the primary motivating factor. One patient said, “*Chemo is the worst thing that you can do to a human being. And when women, when anyone, hears the word chemotherapy, they're scared out of their life. When I told certain people that I had cancer, they said, ‘Oh, I'm so sorry … you have to go through chemo.”* Another said, *“It feels like the drug is going to kill you before the cancer would*.”

Participants identified that patients with prior indirect experience with chemotherapy would be more compelled to participate in a chemotherapy reduction trial compared to those without prior experience. The appeal of reduced toxicity was related to lessened recovery time and higher quality of life during both treatment and survivorship. One advocate explained having a higher “*overall quality of life [and the] ability to maintain as much normalcy as you can*” as a motivating factor. Another said, “*If there is a possibility that you could have less side effects with the same benefit,* *I think any patient would go for that*.”

Trust in the provider was also an important facilitator. One advocate emphasized confidence coming from the relationship with the physician, saying the “*physician is not just looking at data*,” but looking “*at the person and [caring] about the person*.” Other facilitators included increased monitoring, options to increase dosage at any point, contributions to scientific knowledge, and financial and logistical benefits of not having to receive more frequent chemotherapy.

#### 3.1.4 Preferred language to describe the concept of de‐escalation

Only six participants (15%) considered the word “de‐escalation” as positive, saying “*I don't really relate that to cancer treatments and it doesn't* *seem like it fits to me*.” Another exclaimed, “*I'd be like, ‘Hell no!’ Don't de‐escalate. I don't* *want to die*.” Patient‐centered rather than treatment‐centered language was preferred, with positive reactions to words like “personalized,” “customized,” and “tailored.”

#### 3.1.5 Perspectives of COVID‐19’s impact on patient care and de‐escalation

Seventeen participants completed interviews during the COVID‐19 pandemic. Seven (41%) of these respondents initially stated prior to the discussion about the pandemic that they would not have been interested in a less aggressive approach, with one patient expressing uncertainty. However, only one of these patients said she would still prefer aggressive chemotherapy in the setting of the pandemic. Confusion and anxiety were associated with undergoing cancer treatment during a pandemic. “*I think every person in America is stressed. And then, if you add on that diagnosis, you just added this level of complexity … They're anxious because they're in the hospital room … even though they're getting the treatment that they need, there's an anxiety,* *so they're trying to play off this risk of infection and risk of cancer coming back in their minds and it's very complex*.”

### 3.2 Quantitative results

#### 3.2.1 Participants

The survey was sent to 771 female breast cancer patients. A total of 132 opened the recruitment e‐mail. Of those, 115 (87%) clicked through the recruitment e‐mail to view survey information, 110 (83%) consented to the survey, and 91 completed the survey (69%). No notable demographic or clinical differences were found when comparing survey respondents and non‐respondents (Table S1). Of the 91 respondents, median age was 58 years (interquartile range 48–66), 76% were White, and 48% had yearly household incomes <$40,000 (Table 3). The majority (86%) of respondents were initially diagnosed with an early stage breast cancer, 44% had a distant recurrence. Almost two‐thirds (64%) of respondents were on active treatment at the time of survey completion.

**TABLE 3:** Survey respondent demographic and clinical characteristics by interest in de‐escalation trial participation (*N* = 91)

	Total	Interested in de‐escalation trial	Uninterested in de‐escalation trial	Effect size
	*N* = 91	*n* = 52	*n* = 39	
	*n* (%)	*n* (%)	*n* (%)	*V*
Age (median, IQR)	58 (48–66)	60 (51–70)	55 (45–69)	*d* = 0.21
Race				0.02
White	69 (75.8)	39 (56.5)	30 (43.5)	
Other race	22 (24.2)	13 (59.1)	9 (40.9)	
Ethnicity				0.09
Hispanic or Latino	10 (11.0)	7 (70.0)	3 (30.0)	
Non‐Hispanic or Latino	81 (89.0)	45 (55.6)	36(44.4)	
Annual household income				0.08
≥$40,000	47 (51.7)	25 (53.2)	22 (46.8)	
<$40,000	44 (48.4)	27 (61.4)	17 (38.6)	
Marital status				0.23
Single/divorced/widowed	44 (48.4)	20 (45.5)	24(54.6)	
Married	47 (51.7)	32 (68.1)	15 (31.9)	
Employment status				0.17
Working	42 (46.2)	23 (54.8)	19 (45.2)	
Retired	24 (26.4)	16 (66.7)	8 (33.3)	
On disability	18 (19.8)	8 (44.4)	10 (55.6)	
Unemployed/not working	7 (7.7)	5 (71.4)	2 (28.6)	
Health insurance status				0.11
Private	47 (51.7)	29 (61.7)	18 (38.3)	
Medicare	39 (42.9)	20 (51.3)	19 (48.7)	
Medicaid	2 (2.2)	1 (50.0)	1 (50.0)	
Uninsured/unknown	3 (3.3)	2 (66.7)	1 (33.3)	
Breast cancer diagnosis stage				0.18
0	3 (3.3)	1 (33.3)	2 (66.7)	
I	19 (20.9)	11 (57.9)	8 (42.1)	
II	40 (44.0)	21 (52.5)	19 (47.5)	
III	16 (17.6)	10 (62.5)	6 (37.5)	
IV	9 (9.9)	7 (77.8)	2 (22.2)	
Unknown	4 (4.4)	2 (50.0)	2 (50.0)	
Years from diagnosis				0.11
<1–2	30 (33.0)	17 (56.7)	13 (43.3)	
3–4	28 (30.8)	18 (64.3)	10 (35.7)	
5+	33 (36.3)	17 (51.5)	16 (48.5)	
Cancer recurrence	40 (44.0)	24 (60.0)	16 (40.0)	0.05
On active treatment	58 (63.7)	31 (53.5)	27 (46.6)	0.10

Abbreviation: IQR, interquartile range.

#### 3.2.2 De‐escalation trial participation

Many (43%) respondents would be unwilling to participate in a de‐escalation trial. Compared with those willing, these respondents were more often unmarried (55% vs. 32%, *V* = 0.23), younger (median age 55 vs. 60, *d* = 0.21), on disability (56% vs. 40%, *V* = 0.17), or diagnosed with nonmetastatic cancer (45% vs. 22%, *V* = 0.14). In adjusted models, respondents who did versus did not worry about decision regret trended toward disinterest in de‐escalation trial participation (RR 0.51, 95% CI 0.24–1.09).

#### 3.2.3 Facilitators and barriers

**FIGURE 1:**
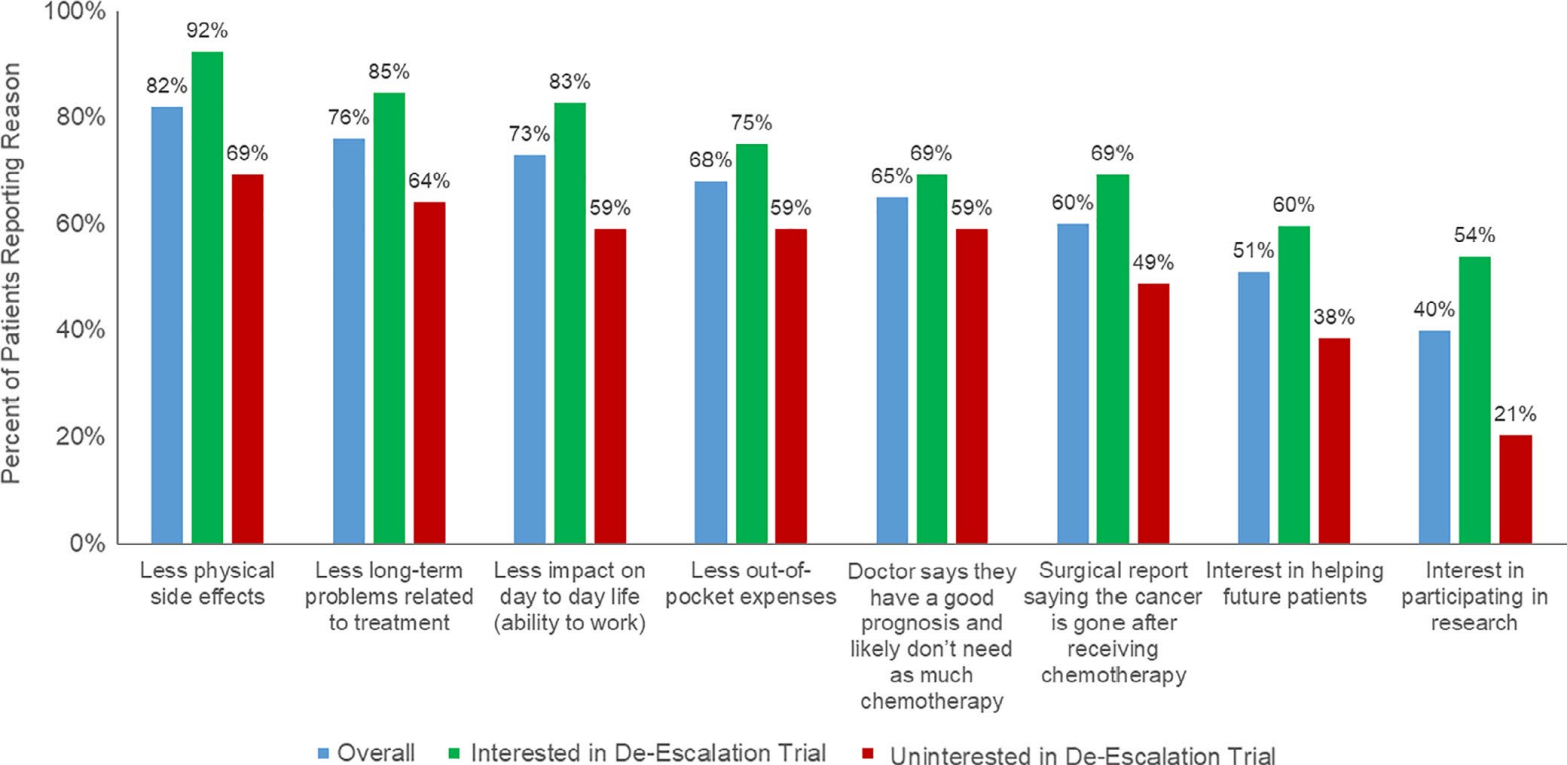
Facilitators to de‐escalation trial participation by interest status (*N* = 91)

**FIGURE 2:**
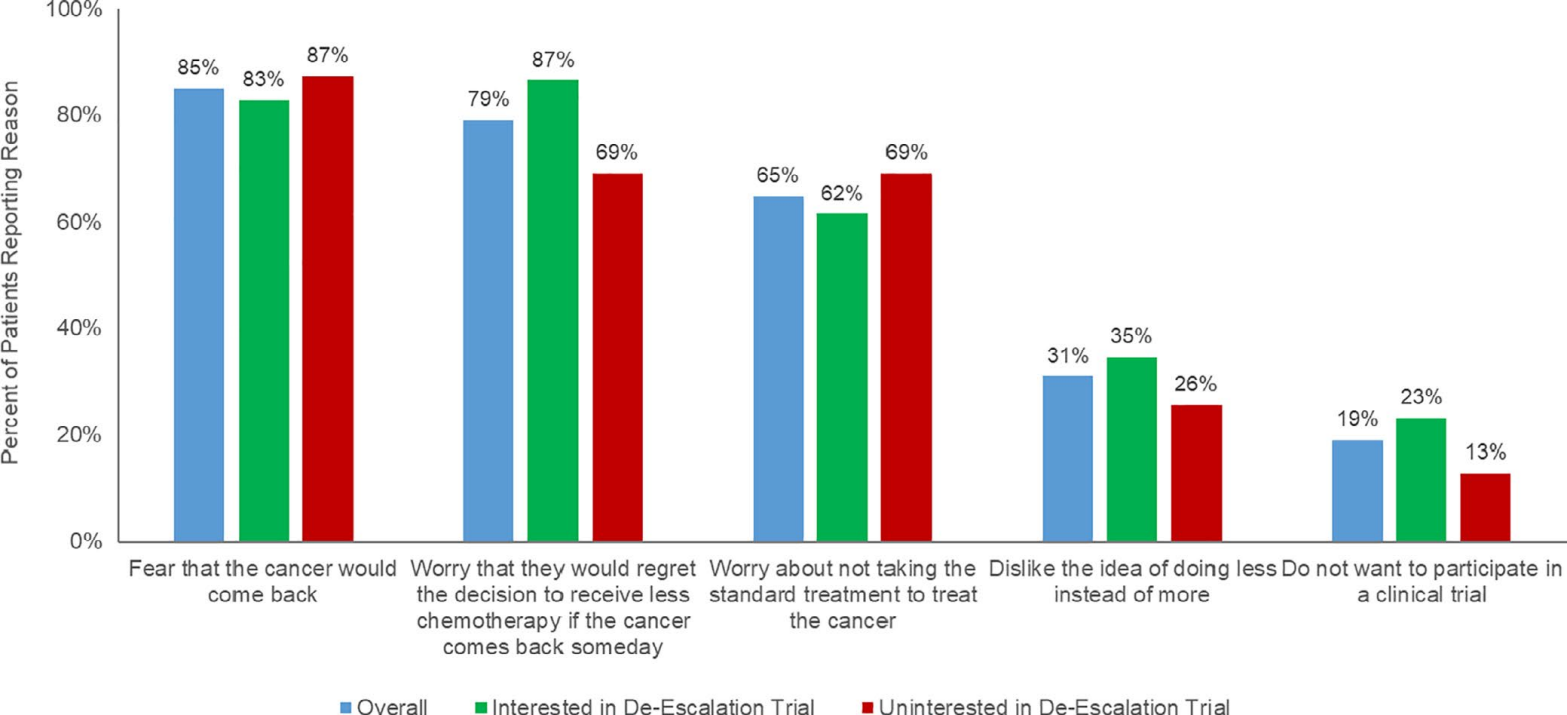
Barriers to de‐escalation trial participation by interest status (*N* = 91)

The most common facilitators to de‐escalation trial participation included decreased physical side effects (82%), less long‐term treatment‐related issues (76%), and lessened impact on daily life (68%; Figure 1). Conversely, the most common barriers to de‐escalation trial participation included fear of cancer recurrence (85%), regret surrounding treatment de‐escalation (79%), and worry about receiving nonstandard treatment (65%; Figure 2). When stratifying by trial participation interest, the greatest facilitator for respondents both uninterested and interested was less physical side effects (69% and 92%, respectively). The greatest barrier for uninterested respondents was fear of recurrence (87%), whereas the greatest barrier perceived by those interested was concern regarding decision regret (87%). Similar results were observed for the subset of patients without recurrence (Figures S1 and S2).

#### 3.2.4 Preferred verbiage to describe de‐escalation

When asked about terminology to describe lessened chemotherapy dosage, no respondent preferred the term “de‐escalation.” The most popular terminology was “lowest effective chemotherapy dose” (53%), followed by “optimization” (22%), and “less chemotherapy” (15%).

### 3.3 Mixed methods interpretation

The qualitative and quantitative data suggest fear is a key driver of reluctance to reduce chemotherapy. Fear included both worry about recurrence and the possibility of decision regret if the cancer returned. Conversely, participants commonly identified avoidance of physical and financial chemotherapy toxicities and lessened impact on daily life as attractive aspects of reduced‐intensity treatment. Respondents in both components identified “de‐escalation” terminology as unfavorable. Interview participants preferred patient‐centered language, such as “personalized” or “optimized,” whereas survey respondents preferred disease‐centered language like “lowest effective chemotherapy dose.”

## 4 DISCUSSION

In this study, many women with breast cancer would have been unwilling to participate in clinical trials testing reduced chemotherapy. Lack of interest was most strongly driven by fear in both the qualitative and quantitative samples. This finding likely represents an exacerbation of known concerns for patients with a history of breast cancer. Distress and fear surrounding cancer recurrence are both common and durable, with 70% of survivors still fearing recurrence 5 years after breast cancer diagnosis.^17^, ^18^ These fears are associated with difficulties in performing daily and social activities, higher depression and anxiety, and lower quality of life.^19^, ^20^ Furthermore, previous literature shows fear of recurrence as more common in younger women, women with children, and women with lower social support.^21^ This was mirrored in our study, with younger women and those with dependents more often preferring intense treatment. Recognition of fears is important when engaging populations who may be particularly vulnerable to adverse psychological outcomes in clinical trials and when applying findings to the general population.

While some reluctance to the concept of de‐escalation existed, patients identified provider recommendation and health‐care team messaging as comforting during treatment decision‐making. In the qualitative analysis, patients repeatedly highlighted trust in their oncologist and reliance on their provider's judgment for optimal treatment selection. Simultaneously, patients wanted clear explanations of the decision rationale and personalization. The importance of shared decision‐making for treatment de‐escalation is therefore underscored. Previous literature has shown that women with early stage breast cancer who engage in shared decision‐making report greater treatment decision satisfaction with less distress, depression, and decision regret.^22^, ^23^ A key component of this engagement is care team language. Few patients in the qualitative analysis and none of the patients in the quantitative analysis preferred the term “de‐escalation,” which is commonly utilized by oncologists designing clinical trials. Patient preference for terms such as “personalized” or “optimized” reflected the overwhelming sentiment for positive messaging from their providers. These findings compliment previous work by Jenkins and colleagues, which found that patients preferred language when explaining clinical trial procedures, such as randomization, that was different than commonly used provider language.^24^ As such, these analyses suggest opportunities for alternate language within the context of shared decision‐making that will help patients both better understand the concept of reducing treatment intensity and increase comfort with treatment decisions.

The COVID‐19 pandemic shifted opinions of several participants, with some transitioning from a preference for more chemotherapy to avoidance of chemotherapy entirely. Given the pandemic is expected to last for months to years, the threat of COVID‐19 may permanently shift patient and provider willingness to consider de‐escalation strategies both during and post‐pandemic. This opens a window of opportunity to study more tailored or less treatment for patients and to initiate communication with patients about their cancer‐related risk in relation to COVID‐19 exposure and adverse outcomes. Although this study suggests that concerns related to COVID‐19 may drive patient care access, further work will be needed to explore how newly diagnosed patients are integrating this information into their care and how views change as the pandemic progresses.

This study has several limitations. The interview sample was exclusively female patients with breast cancer, which may not reflect the views of males or patients with other cancers. However, fear of recurrence is common across cancer types.^25^, ^26^, ^27^ This evaluation focused on chemotherapy and may not reflect views of other de‐escalation approaches, such as decreased radiation or surgery. Additionally, the sample size was limited by patients who did not open the e‐mail and thus could not complete the survey, which could lead to bias. However, demographic characteristics were similar between respondents and nonrespondents. The small sample size may have influenced the modest differences in barriers and facilitators reported between those of would and would not be interested in clinical trials. Furthermore, the survey included patients who sought financial assistance for medical expenses and predominantly had lower income levels, thus their perspectives may not reflect those of a population with more financial resources. We did not characterize the severity of side effects experienced by participants, which could influence perspectives. Finally, the survey was not designed to explore decision‐making tradeoffs such as toxicity avoidance, shorter treatment course, or other beneficial aspects of de‐escalation. Patient interviews were conducted from a single site, which may limit generalizability. However, themes were consistent with those identified by the patient advocates and the survey respondents, which were nationally representative samples. Although the interviewer was not the oncologist responsible for treatment decisions, patients may have been less forthcoming with a physician than with another member of the research team, resulting in bias. This limitation was felt to be balanced by gains due to the interviewer‘s clinical expertise resulting in a unique ability to probe participants, yielding greater depth in data collected. We did not characterize differences between Black and White patients due to limited sample size, although this will be examined in a subsequent, larger study. The survey did not utilize a validated tool to evaluate patient perspectives on de‐escalation; rather, it quantified initial observations identified from early qualitative interviews. Additional evaluation using validated tools to assess recurrence distress, anxiety, and decision regret should be included as patient‐reported outcomes within future clinical trials.

## 5 CONCLUSION

This mixed methods analysis revealed fear surrounding recurrence as a major barrier to participation in a clinical trial testing chemotherapy de‐escalation in women with breast cancer. Conversely, avoidance of physical and financial chemotherapy toxicities and lessened impact on daily life were attractive aspects of reduced‐intensity treatment among patients vulnerable to treatment‐related financial toxicity. Additional research is needed to understand how best to engage patients in decision‐making about reducing the intensity of treatment, particularly in the setting of a global pandemic.

## AUTHOR CONTRIBUTION

Conceptualization: GR, CW, LW. Methodology: GR, CA, LW. Data curation and analysis: all. Investigation: all. Writing—original draft: GR. Writing—review and editing: all. Supervision, project administration, funding acquisition: GR.

## DISCLOSURES

Dr. Rocque received research funding from Genentech, Pfizer, and Carevive and consulting fees for Genentech and Pfizer.

## ETHICS

This study was approved by the University of Alabama at Birmingham Institutional Review Board.

## Supporting information

Supplementary Material

## Data Availability

The data that support the findings of this study are available from the corresponding author upon reasonable request.
