# Supplementary material for: Patient perspectives on chemotherapy de‐escalation in breast cancer
Source: Cancer Med. 2021 May 1;10(10):3288–98. doi: 10.1002/cam4.3891 (PMC8124110; doi:10.1002/cam4.3891)
Supplement: Supplementary file 1 — Supplementary Material [file CAM4-10-3288-s001.zip › cam43891-sup-0001-FigS2-3.docx]

Supplemental Figure 2:

Supplemental Figure 3:
