# Supplementary material for: Patient perspectives on chemotherapy de‐escalation in breast cancer
Source: Cancer Med. 2021 May 1;10(10):3288–98. doi: 10.1002/cam4.3891 (PMC8124110; doi:10.1002/cam4.3891)
Supplement: Supplementary file 1 — Supplementary Material [file CAM4-10-3288-s001.zip › cam43891-sup-0002-TableS1.docx]

Supplemental Table 1. Demographics and clinical characteristics of Patient Advocate Foundation patients sent recruitment e-mail (N=771).

|  | n (%) |
| --- | --- |
| Age (median, IQR) | 59 (42-76) |
| Race |  |
| White | 514 (66.7) |
| Non-White | 213 (27.6) |
| Unknown | 44 (5.7) |
| Ethnicity |  |
| Hispanic or Latino | 63 (8.2) |
| Non-Hispanic or Latino | 664 (86.1) |
| Unknown | 44 (5.7) |
| Annual household income |  |
| ≥$40,000 | 285 (63.0) |
| <$40,000 | 486 (37.0) |
| Marital status |  |
| Single/Divorced/Widowed | 391 (50.7) |
| Married | 372 (48.3) |
| Unknown | 8 (1.0) |
| Employment status |  |
| Working | 253 (32.8) |
| Retired | 265 (34.4) |
| On disability | 130 (16.9) |
| Unemployed / Not working | 101 (13.1) |
| Unknown | 22 (2.9) |
| Health insurance status |  |
| Private | 364 (47.2) |
| Medicare | 360 (46.7) |
| Medicaid | 16 (0.0) |
| Uninsured / Unknown | 31 (0.0) |
| Breast cancer diagnosis stage |  |
| IV | 37 (4.8) |
| Unknown | 734 (95.2) |
| On active treatment | 771 (100.0) |
