# Supplementary material for: Patient perspectives on chemotherapy de‐escalation in breast cancer
Source: Cancer Med. 2021 May 1;10(10):3288–98. doi: 10.1002/cam4.3891 (PMC8124110; doi:10.1002/cam4.3891)
Supplement: Supplementary file 1 — Supplementary Material [file CAM4-10-3288-s001.zip › cam43891-sup-0003-Supinfo.docx]

Supplemental Document 1: Example explanation of de-escalation concept

“We are currently planning a study, which tests the ability to decrease the amount of chemotherapy given to patients with breast cancer. This may result in fewer short-term or permanent side effects from chemotherapy. For example, patients might get one chemotherapy instead of two at first. We can then see how they respond to treatment by looking at how much cancer remains when they later have breast surgery. If they have no cancer still in the breast, they don’t get more chemotherapy. If all the cancer wasn’t gone, we could give more chemotherapy after surgery. We will evaluate if those patients who got less than the usual amount of chemotherapy have recurrence rates that are similar to rates reported in for patients not in a study who got the usual amount of chemotherapy.”

Supplemental Document 2: Survey questions

We are interested in how women with breast cancer think about decisions made by patients and doctors related to the amount of chemotherapy they may receive.

We know that cancer is less likely to come back for women whose cancer in the breast (or underarm lymph nodes) disappears in response to the chemotherapy given before surgery. Research studies are being planned that test whether lower doses of chemotherapy can do just as well at curing breast cancer as what is currently given. Studying whether we can give less chemotherapy is an important research question, because it may result in fewer short-term or permanent side effects for breast cancer survivors.

1. If you were asked to participate in a study testing lower doses of chemotherapy than the usual amount of chemotherapy, would you agree to participate?
   1. Yes
   2. No
2. Do you think any of the following reasons would make patients more interested in receiving less chemotherapy? Check all that apply
   - Less physical side effects
   - Less out-of-pocket expenses
   - Less long-term problems related to treatment
   - Less impact on day to day life (ability to work)
   - Surgical report saying the cancer is gone after receiving chemotherapy
   - Doctor says they have a good prognosis and likely don’t need as much chemotherapy
   - Interest in participating in research
   - interest in helping future patients
   - Other_______

3.     What do you think would make patients not want to participate? (Check all that apply)

- - Fear that the cancer would come back
  - Worry that they would regret the decision to receive less chemotherapy if the cancer comes back someday
  - Worry about not taking the standard treatment to treat the cancer
  - Do not want to participate in a clinical trial
  - Dislike the idea of doing less instead of more
  - Other ________

4.     How worried would you be during or after treatment if you heard your friends with similar cancer received more treatment than you?

1-10 from not worried at all to extremely worried

5.     We are interested in what words we can use to describe this approach. What words would you use to describe giving less chemotherapy to patients who we don’t think need a high dose?

- - De-escalation
  - De-intensification
  - Optimization
  - Less chemotherapy
  - Lowest effective chemotherapy dose
  - Other

6.     Please comment here if there is any other feedback you would like to share:____________________
